# Supplementary material for: Anatomical and functional connectivity support the existence of a salience network node within the caudal ventrolateral prefrontal cortex
Source: eLife. 2022 May 5;11:e76334. doi: 10.7554/eLife.76334 (PMC9106333; doi:10.7554/eLife.76334)
Supplement: Supplementary file 1. [file elife-76334-supp1.pptx]

## Slide 1
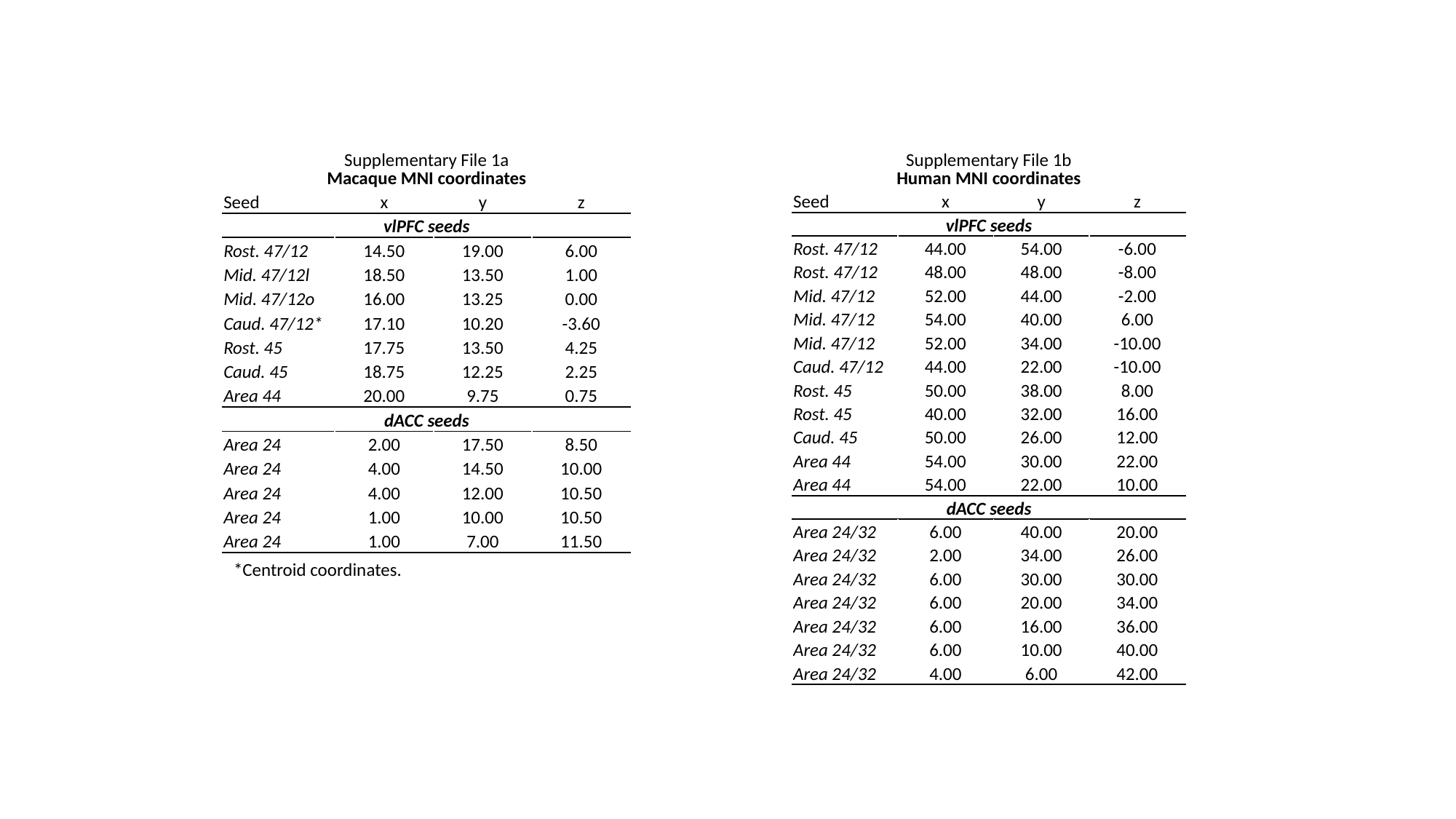

Supp. Tables
| Supplementary File 1a Macaque MNI coordinates | | | |
| --- | --- | --- | --- |
| Seed | x | y | z |
| vlPFC seeds | | | |
| Rost. 47/12 | 14.50 | 19.00 | 6.00 |
| Mid. 47/12l | 18.50 | 13.50 | 1.00 |
| Mid. 47/12o | 16.00 | 13.25 | 0.00 |
| Caud. 47/12\* | 17.10 | 10.20 | -3.60 |
| Rost. 45 | 17.75 | 13.50 | 4.25 |
| Caud. 45 | 18.75 | 12.25 | 2.25 |
| Area 44 | 20.00 | 9.75 | 0.75 |
| dACC seeds | | | |
| Area 24 | 2.00 | 17.50 | 8.50 |
| Area 24 | 4.00 | 14.50 | 10.00 |
| Area 24 | 4.00 | 12.00 | 10.50 |
| Area 24 | 1.00 | 10.00 | 10.50 |
| Area 24 | 1.00 | 7.00 | 11.50 |
| Supplementary File 1b Human MNI coordinates | | | |
| --- | --- | --- | --- |
| Seed | x | y | z |
| vlPFC seeds | | | |
| Rost. 47/12 | 44.00 | 54.00 | -6.00 |
| Rost. 47/12 | 48.00 | 48.00 | -8.00 |
| Mid. 47/12 | 52.00 | 44.00 | -2.00 |
| Mid. 47/12 | 54.00 | 40.00 | 6.00 |
| Mid. 47/12 | 52.00 | 34.00 | -10.00 |
| Caud. 47/12 | 44.00 | 22.00 | -10.00 |
| Rost. 45 | 50.00 | 38.00 | 8.00 |
| Rost. 45 | 40.00 | 32.00 | 16.00 |
| Caud. 45 | 50.00 | 26.00 | 12.00 |
| Area 44 | 54.00 | 30.00 | 22.00 |
| Area 44 | 54.00 | 22.00 | 10.00 |
| dACC seeds | | | |
| Area 24/32 | 6.00 | 40.00 | 20.00 |
| Area 24/32 | 2.00 | 34.00 | 26.00 |
| Area 24/32 | 6.00 | 30.00 | 30.00 |
| Area 24/32 | 6.00 | 20.00 | 34.00 |
| Area 24/32 | 6.00 | 16.00 | 36.00 |
| Area 24/32 | 6.00 | 10.00 | 40.00 |
| Area 24/32 | 4.00 | 6.00 | 42.00 |
*Centroid coordinates.
